# Treatment of Graf Type IIa Hip Dysplasia: A Cut-off Value for Decision Making

**DOI:** 10.4274/balkanmedj.2017.1150

**Published:** 2018-11-15

**Authors:** Fuat Bilgili, Yavuz Sağlam, Süleyman Bora Göksan, Önder Murat Hürmeydan, Fevzi Birişik, Mehmet Demirel

**Affiliations:** 1 Department of Orthopedics and Traumatology, İstanbul University İstanbul School of Medicine, İstanbul, Turkey; 2 Department of Orthopedics and Traumatology, Biruni University School of Medicine, İstanbul, Turkey

**Keywords:** Decisioion making, Graf type IIa, Hip dysplasia, Ultrasonography

## Abstract

**Background:**

The rate of spontaneous normalization in type IIa hips is reported to be high, whereas dysplsia persists or worsens in 5%-10% of cases.

**Aims:**

To evaluate the natural course of type IIa hips using Graf’s own perspective of physiological immaturity and maturational deficit.

**Study Design:**

A single center, retrospective cohort study.

**Methods:**

This was an institutional review board-approved retrospective review of all patients diagnosed with type IIa hip dysplasia at a single institution from 2012 to 2014. All patients included in the study had hip ultrasonography at about 6 weeks and 3 months of age. To assess reliability in α and β angles, ultrasonography measurements were carried out on the same image individually by all observers. The α and β angles were used as the main outcome measurements to evaluate hip maturation at the last follow-up. A receiver operating characteristics curve was drawn at the 3 month ultrasonography to evaluate the cut-off values for α and β angles for persistent dysplasia.

**Results:**

Sixty-four patients and 88 affected hips (63% unilateral and 37% bilateral) were included. The mean age at diagnosis was 6.4±2.7 weeks. Fifty-four hips were type IIa(+) (physiologically immature) and 34 hips were type IIa(-) (maturational deficit) at the initial ultrasonography evaluation. Improvement to type I was seen in 52 type IIa(+) and 17 type IIa(-) hips. Receiver operating characteristic analyses showed that patients do well if the α angle was >55° (area under the curve: 0.86; p<0.001 for the left hip and area under the curve: 0.72; p=0.008 for the right hip).

**Conclusion:**

The cut-off α angle value of 55° on initial ultrasonography should be considered to prevent future dysplasia. An α angle <55° on the initial ultrasonography was an independent predictor of worsening sonographic findings.

Developmental dysplasia of the hip (DDH) is one of the most common musculoskeletal problems in newborns (1). The incidence of DDH varies from 0.5% to 30% according to geographical region and ethnicity (2). The mean incidence is 2.5% in Turkey. Treatment is easier and complications are less likely to occur if DDH is diagnosed early (3). Hip ultrasound, which was described by Graf, has been used to evaluate DDH in children since the early 1980s (4,5). Ultrasound provides detailed visualization of the cartilaginous anatomy of the hip that is not revealed by plain X-rays and allows early detection of DDH (6,7). Hips with a slightly shallow acetabulum and rounded bony rim before 3 months of age are considered developmentally immature and are classified as Graf type IIa (5). The α angle is 50-59°, whereas the β angle is 55-77° (8). These hips are clinically reduced and stable (8). The prevalence of type IIa hips ranges from 10% to 45% depending on the age of the population (9,10). The rate of spontaneous normalization in type IIa hips is reported to be 90-97%, whereas dysplasia persists or worsens in 3-10% of cases (7,10,11). Type IIa hips have been divided into two subtypes to differentiate hips that tend to deteriorate; type IIa(+) (physiologically immature) and type IIa(-) (maturational deficit), which should be decided according to the age in weeks and the α angle (Figure 1) (7,8). Type IIa(+) hips are still within the acceptable limits for age (7). If a type IIa hip does not reach the minimum linear maturation rate, then it is called a type IIa(-) hip (7). Management of type IIa(-) hips remains controversial and these hips always carry the risk of either overtreatment or development of true hip dysplasia (7). In our study, we addressed whether all Graf type IIa hips should be treated and evaluated the natural course of type IIa hips using Graf’s own perspective of physiological immaturity and maturational deficit.

## MATERIALS AND METHODS

This was an Institutional Review Board-approved retrospective review of all patients diagnosed with type IIa hip dysplasia at a single institution from 2012 to 2014. The Graf technique was used at all ultrasound evaluations (5). Patients with a diagnosis of congenital coxa vara, congenital short femur, skeletal dysplasia, or metabolic bone disease were excluded. Patients able to return to the 3 month ultrasound follow-up were included. Hips that progressed to Graf type IIb at the 3 month ultrasound evaluation were treated with a hip abduction orthosis. Informed consent was obtained from all parents of children included in the study.

### Radiographic measurements and intra- and inter-observer reliability

All patients had hip ultrasound at about 6 weeks and 3 months of age. The ultrasound parameters were measured by three independent observers (Y.S., O.M.H., and F.B.). Inter- and intra-observer reliability for each measurement was assessed using intra-class correlation coefficients (ICC) calculated from two sets of repeat measurements on a subset of 30 sonograms at least 1 week apart for each observer. Agreement was considered excellent for ICC >0.80, very good for 0.70-0.80, good for 0.60-0.70, fair for 0.40-0.60, and poor for <0.40. Intra- and inter-observer reliability of the α angle measurements were very good (0.791 and 0.722, respectively). However, the β angle measurements showed good intra- and fair inter-observer reliability (0.661 and 0.537, respectively). The α and β angles were used as the main outcome measurements to evaluate hip maturation at the last follow-up.

### Statistical analysis

The statistical analysis was performed using SPSS software (SPSS Inc., Chicago, IL, USA). A receiver operating characteristics (ROC) curve was drawn at the 3 month USG to evaluate the cutoff values of the α and β angles.

## RESULTS

**Figure 2:**
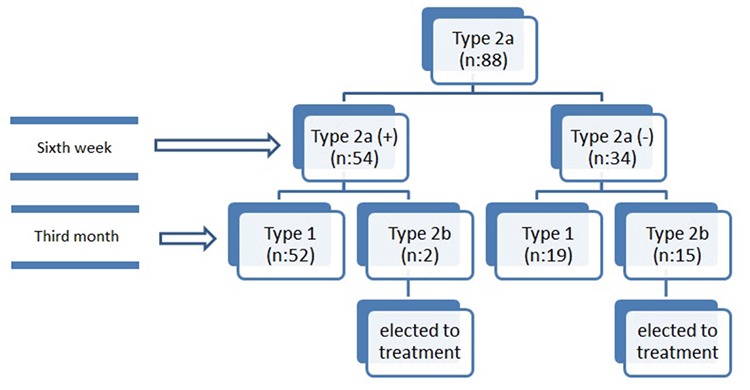
Initial and follow-up ultrasound measurements and typology of the hips.

Sixty-four patients (46 females an 18 males) with 88 affected hips (63% unilateral and 37% bilateral) were included in this study. Mean age at diagnosis was 6.4±2.7 weeks. Fifty-four hips were classified as type IIa(+), and 34 hips were classified as type IIa(-) at the initial ultrasound evaluation. After an observation period of 6 weeks, improvement to type I was seen in 52 type IIa(+) and 17 type IIa(-) hips. According to the 3 month ultrasound measurements, a type IIb transition was seen in only two hips in the type IIa(+) group and in 15 hips in the type IIa(-) group (Figure 2).

**Figure 3:**
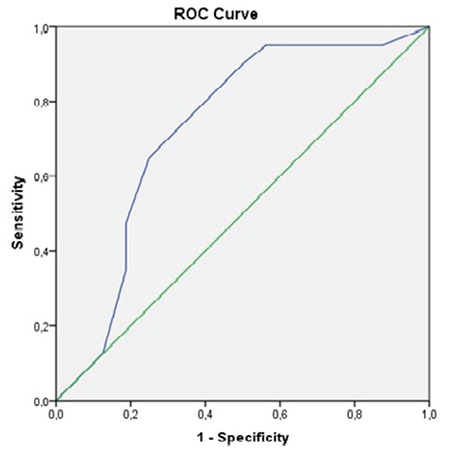
Receiver operating characteristic curve of the α values for the right hip. *ROC: Receiver operating characteristic*

**Figure 4:**
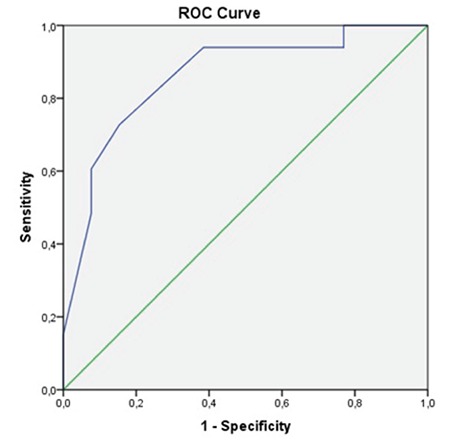
Receiver operating characteristic curve of the α values for the left hip. *ROC: Receiver operating characteristic*

**Figure 5:**
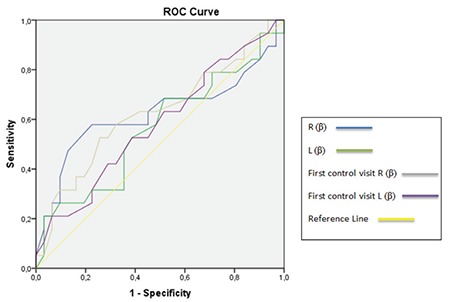
Receiver operating characteristic curve of the β values for the left and right hips. *ROC: Receiver operating characteristic*

ROC analyses showed that patients did well if the α angle was >55° on their first ultrasound evaluation (Table 1, Figure 3, 4, 5). Patients who had an α angle <55° were more likely to develop an immature hip joint at 3 months of age, regardless of the age of the infant at the initial USG [area under the curve (AUC): 0.86; p<0.001 for the left hip and AUC: 0.72; p=0.008 for the right hip]. According to the ROC analyses, there was no cut-off value for the β angle between the treated and untreated groups (Figure 5). No femoral palsies or dislocations were observed related to the use of a harness. All hips improved to type I, and none of the patients needed casting or surgical intervention at the 1-year follow-up.

## DISCUSSION

The high rate of spontaneous improvement reported for DDH (>80%) has led to a considerable dilemma regarding the approach to be used for screening positive infants (6). It has been argued that ultrasound screening techniques lead to the diagnosis of a higher number of DDH cases compared with a physical examination (Ortolani and Barlow clinical tests) and that this can increase the risk of treatment being prescribed unnecessarily (6). However, an early diagnosis and treatment of DDH, made possible by ultrasound screening, can lead complete recovery. Therefore, we evaluated the natural course of type IIa hips using Graf’s own perspective of physiological immaturity and maturational deficit.

In Graf’s original study, type IIa is divided into two subtypes of type IIa(+) (physiologically immature) and type IIa(-) (maturational deficit), which does not reach the minimum linear maturation rate by age in weeks (5,12,13).

The rate of spontaneous resolution has been reported to be about 95% in type IIa hips (1,11,14,15,16). Kosar et al. (16) reported that 5.6% of type IIa hips worsen sonographically at follow-up. Central nervous system anomalies, instability, and unilateral type IIa hips were predictors of worsening in their series (16). In our study, a type IIb transition was seen in 19.3% (17 of 88 hips) of type IIa hips at the 3 month ultrasound examination. This transition was more likely to occur in type IIa(-) patients according to the initial α angle measurements. Omeroğlu et al. (7) recommend paying more attention to type IIa hips in newborn girls, as they found a lower rate of spontaneous normalization in newborn girls than in boys. Those authors did not provide the overall rate of spontaneous normalization in type IIa hips at the end of the third month, as they preferred to treat rather than to follow the type IIa(-) hips (7). Graf also recommends treating type IIa(-) hips with an orthosis (8,12). In contrast to the literature, we report a cutoff α angle value that can be used as a guide for pediatric orthopedic surgeons to predict which hips may worsen. Our study showed that an α angle <55° at week 6 of life needs treatment and confirms the value of subtyping type IIa hips as described by Graf (8,12). The skill of physicians in identifying this disorder is controversial in ultrasound-based studies. In our study, there may have been some type IIa cases that were sonographically under-diagnosed or over-diagnosed on the initial ultrasound. All measurements were done by three independent observers to avoid under- or over-diagnosis, eliminate the bias of the physician, and to assess intra- and inter-observer reliability of the α and β angles. The limitations of this study were mostly caused by its retrospective design. The number of patients was comparatively small, and the duration and modality of treatment were not compared. Prospective controlled studies comparing the effects of observation and orthotic treatment on long-term outcomes of the type IIa hips may be beneficial.

In conclusion, ultrasound follow-up is necessary for all Graf type IIa hips; however, treatment is not necessary for all Graf type IIa hips. An α angle <55° on the initial ultrasound was an independent predictor for worsening, and this cutoff value should be considered to prevent future dysplasia. These patients usually require treatment with an orthosis, along with a more careful follow-up.

## Figures and Tables

**Table 1:**
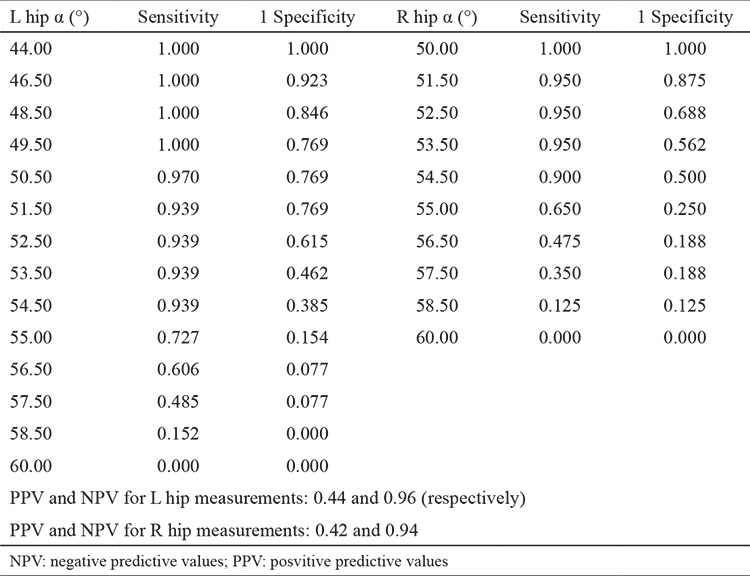
Sensitivity and specificity of the initially measured α values to predict maturation of both hips at month 3 (The smallest cutoff value is the minimum observed test value minus one, and the largest cutoff value is the maximum observed test value plus one. All other cutoff values are the averages of two consecutive ordered observed test values)

**Figure 1:**
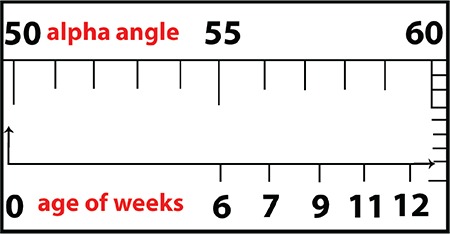
Definition of type IIa(+) hips; The alpha angle should be ≥55° in week 6, ≥56° in week 7, >57° in week 9, >58° in week 11 and ≥60° in week 12. If the angle is >50° but it was not classified as type IIa(+) at these weeks of age, it should be classified as type IIa(-).
